# Clinical manifestations, etiology and outcome of sepsis in pediatric patients admitted to the ICU

**DOI:** 10.1186/cc12918

**Published:** 2013-11-05

**Authors:** Taís C São Pedro, André M Morcillo, Emilio CE Baracat

**Affiliations:** 1 Department of Pediatrics, State University of Campinas, UNICAMP, Campinas, SP, Brazil

## Background

Sepsis still represents the leading cause of mortality among children and its etiology changes according to age, immune status and geographic location [1-4]. Prevention of this disease has key role in reducing morbidity and mortality and includes development and application of vaccines [5-7]. In 2010, pneumococcal and meningococcal C vaccines were introduced in the basic immunization schedule in Brazil. The application of these may already be influencing the etiologic profile of sepsis in childhood [7]. The evaluation of this profile, as well as the clinical manifestations and course of sepsis in the post vaccine, becomes essential for better clinical decision and effective therapeutic approach in hospitalized patients. The objective was to determine clinical manifestations, etiology and outcome of sepsis in patients admitted to a pediatric ICU.

## Materials and methods

A retrospective cohort study, by collecting data from medical records of patients diagnosed with sepsis admitted to the pediatric ICU of Hospital Municipal Dr. Mario Gatti, Campinas, SP, from January 2011 to December 2012. The variables studied were: age, sex, vaccination schedule, etiologic agent identified in cultures and clinical outcome.

## Results

Eighty-seven patients were included in the study (56 male, 31 female) with a mean hospital stay of 8.16 days, vasoactive drug use of 2.82 days and 5.33 days of mechanical ventilation. In total, 57/87 cultures were negative. Among the positive, the majority (21/30) was collected less than 48 hours after admission and the most frequent etiologies were: Gram-negative bacteria (10), *Staphylococcus aureus *(7) and *Neisseria meningitidis *(4). Two cultures were positive for *Streptococus *pyogenes and only one for *Streptococcus pneumoniae*. Twenty-four (16.1%) patients died. Mortality was higher in patients with incomplete immunization (*P *= 0.047). Among the cases with meningococcal etiology, 3/4 were not vaccinated.

## Conclusions

The clinical group of patients diagnosed with sepsis showed short time of hospitalization, use of vasoactive drugs and mechanical ventilation. Mortality was high and higher in the group of patients with incomplete immunization. Among the causative agents, it was predominantly Gram-negative bacteria and *S. aureus*, no vaccine-preventable etiologies.
